# Salience network structural integrity predicts executive impairment in alcohol use disorders

**DOI:** 10.1038/s41598-018-32828-x

**Published:** 2018-09-27

**Authors:** Caterina Galandra, Gianpaolo Basso, Marina Manera, Chiara Crespi, Ines Giorgi, Giovanni Vittadini, Paolo Poggi, Nicola Canessa

**Affiliations:** 1 Scuola universitaria superiore IUSS, Pavia, 27100 Italy; 2 Cognitive neuroscience laboratory, ICS Maugeri, Pavia, 27100 Italy; 3 LabNIT, ICS Maugeri, Pavia, 27100 Italy; 4 University of Milano-Bicocca, Milan, 20126 Italy; 5 Clinical psychology unit, ICS Maugeri, Pavia, 27100 Italy; 6 Functional rehabilitation unit, ICS Maugeri, Pavia, 27100 Italy; 7 Radiology unit, ICS Maugeri, Pavia, 27100 Italy

## Abstract

The neural bases of cognitive impairment(s) in alcohol use disorders (AUDs) might reflect either a global brain damage underlying different neuro-cognitive alterations, or the involvement of specific regions mostly affected by alcohol neuro-toxic effects. While voxel-based-morphometry (VBM) studies have shown a distributed atrophic pattern in fronto-limbic and cerebellar structures, the lack of comprehensive neuro-cognitive assessments prevents previous studies from drawing robust inferences on the specificity of the association between neuro-structural and cognitive impairments in AUDs. To fill this gap, we addressed the neuro-structural bases of cognitive impairment in AUDs, by coupling VBM with an in-depth neuropsychological assessment. VBM results highlighted a diffuse pattern of grey matter reduction in patients, involving the key-nodes of the meso-cortico-limbic (striatum, hippocampus, medial prefrontal cortex), salience (insular and dorsal anterior cingulate cortex) and executive (inferior frontal cortex) networks. Grey matter density in the insular and anterior cingulate sectors of the salience network, significantly decreased in patients, explained almost half of variability in their defective attentional and working-memory performance. The multiple cognitive and neurological impairments observed in AUDs might thus reflect a specific executive deficit associated with the selective damage of a salience-based neural mechanism enhancing access to cognitive resources required for controlled cognition and behaviour.

## Introduction

Alcohol use disorders (AUDs) are associated with adverse physical, psychological and social consequences, leading to 3.3 million deaths each year^1^. While AUD patients’ profile of cognitive impairment^2–5^ has been associated with the specific susceptibility of frontal cortex^6^, neuroimaging studies highlighted an extensive damage fitting with the *diffuse brain hypothesis*^7^. Voxel-based morphometry (VBM) studies have indeed shown, in AUDs, an atrophic pattern involving lateral prefrontal cortex, anterior (ACC) and posterior cingulate cortex, insular-opercular cortex, thalamus, hippocampus and striatum^8,9^. This evidence supports the hypothesis that the cognitive alterations observed in addictions involve a cortico-striatal-thalamic circuit including the key-nodes of the executive and salience networks^10^, with the latter mediating the switch between rest and controlled cognition and behaviour^10–12^.

Since abstinence appears to reverse behavioural and neuro-structural damage^13^, treatment protocols would benefit from a detailed characterization of the neural correlates of cognitive impairment(s). Previous studies have addressed a relationship between atrophy and performance in different domains, e.g. executive functioning^14,15^, cognitive control^16,17^ and memory^18^. However, their inconsistent results^14,15^, and the lack of comprehensive neuropsychological assessments, highlight the need of further evidence on the neural bases of cognitive impairment(s) in AUDs.

We pursued this goal at the neuro-structural level, by coupling VBM with an extensive neuro-cognitive assessment. Unlike previous studies we adopted a multivariate approach to investigate, in AUDs, defective cognitive domains transcending specific tasks. We expected impaired performance to be predicted by GM density in regions within the executive or salience networks.

## Results

### Cognitive impairments in alcoholic patients

There was no significant group difference concerning age, education or nicotine consumption (Table 1). Compared with controls, patients displayed significantly worse performance in the ENB global score, and in different sub-scores: immediate recall, interference memory-10”, TMT-A and B, overlapping pictures and clock-drawing (Table 2a). TMT performance is indexed by the total time to completion^19^, i.e. shorter time corresponds to better performance. Age was negatively related to performance in TMT-A (i.e. positively correlated with response time; r = 0.46, p = 0.003), and both immediate and delayed recall (both r = −0.31, p = 0.046). Except for the clock-drawing task, even when controlling for age or education via ANCOVA we confirmed, in patients vs. controls, worse performance in all these tasks (Table 2b,c). A correction for multiple comparisons based on False Discovery Rate (FDR) confirmed the latter evidence, with the only exception of the immediate recall score which showed a marginal trend (p-corrected = 0.058).

**Table 1:** Demographics and alcohol use variables.

	mean HC (n = 18)	mean AUD (n = 23)	SD HC	SD AUD	DF	T-score	p-value
Group comparisons							
*Demographic variables*							
Age (years)	44.833	45.696	8.860	7.824	39	−0.330	0.371
Education (years)	10.111	10.000	2.784	2.629	39	0.131	0.448
Nicotine consumption (yes/no)	6/12	18/5					0.184
*Alcohol use variables*	Mean all patients	SD all patients	Mean females	SD females	Mean males	SD males	p-value
Duration of alcohol use (years)	10.8	7.21	11.89	7.11	10.11	7.48	0.576
Average daily alcohol dose (UA)	14.48	6.55	14.94	5.92	14.18	7.12	0.791

In the top sector of the table, the mean and standard deviation (SD) of demographic variables and nicotine consumption are reported for healthy controls (HC) and alcoholic patients (AUD), alongside the results of group comparisons with two-sample t-tests. In the bottom part, disease duration and average daily alcohol usage are reported both for the whole patient sample and separately for males and females, alongside the results of gender comparisons with two-sample t-tests. DF: degrees of freedom, UA: Units of Alcohol.

**Table 2:** Neuro-cognitive performance.

*Neuro-cognitive variables*	mean HC	mean AUD	SD HC	SD AUD	DF	T-score/U*	p-value	FDR p-value
(a) Group comparison (two-sample t-test)								
**ENB2 global score**	84.111	77.913	7.395	8.096	39	2.526	**0.008**	**0.034**
Digit span*	5.778	5.739	1.166	1.214	39	0.328*	0.371	0.437
**Immediate recall**	15.222	12.826	4.570	4.271	39	1.729	**0.046**	0.111
Delayed recall	20.333	18.826	5.041	5.131	39	0.941	0.176	0.374
**Interference memory 10”**	7.611	6.348	1.614	1.945	39	2.220	**0.016**	0.054
Interference memory 30”	6.944	6.522	2.014	2.233	39	0.628	0.267	0.428
**Trail Making test A**	19.167	29.130	5.448	5.857	39	−5.572	**<0.0001**	**<0.0001**
**Trail Making test B**	68.556	89.783	21.786	41.642	39	−1.959	**0.029**	0.082
Token test*	4.972	4.935	0.118	0.172	39	0.394*	0.347	0.437
Phonemic fluency*	12.721	12.635	3.075	3.366	39	0.085*	0.467	0.467
Abstract verbal reasoning*	5.667	5.609	0.970	0.839	39	0.276*	0.391	0.437
Cognitive estimation*	4.722	4.739	0.461	0.541	39	−0.250*	0.401	0.437
**Overlapping figures**	36.944	31.174	5.514	5.606	39	3.294	**0.001**	**0.008**
Copy drawing*	1.833	1.652	0.383	0.573	39	0.775*	0.219	0.413
Spontaneous drawing	1.889	1.739	0.323	0.541	39	0.591*	0.277	0.428
Clock drawing*	9.389	8.304	2.349	2.406	39	2.706*	**0.003**	**0.017**
Praxic abilities*	6.000	5.957	0.000	0.209	39	0.223*	0.412	0.437
(b) Group comparison controlling for age (ANCOVA)								
*Neuro-cognitive variables*	DF	F	p-value	FDR p-value				
**ENB2 global score**	1,38	6.169	**0.009**	**0.021**				
**Immediate recall**	1,38	2.845	**0.050**	0.058				
**Interference memory 10”**	1,38	4.700	**0.018**	**0.031**				
**Trail Making test A**	1,38	41.300	**<0.0001**	**<0.0001**				
**Trail Making test B**	1,38	3.636	**0.032**	**0.044**				
**Overlapping figures**	1,38	10.440	**0.002**	**0.007**				
Clock drawing*	1,38	1.952	0.085	0.085				
(c) Group comparison controlling for education (ANCOVA)								
*Neuro-cognitive variables*	DF	F	p-value	FDR p-value				
**ENB2 global score**	1,38	6.914	**0.006**	**0.014**				
**Immediate recall**	1,38	2.895	**0.0485**	0.056				
**Interference memory 10”**	1,38	5.18	**0.0145**	**0.021**				
**Trail Making test A**	1,38	30.42	**<0.0001**	**<0.0001**				
**Trail Making test B**	1,38	3.729	**0.0155**	**0.021**				
**Overlapping figures**	1,38	10.86	**0.001**	**0.003**				
Clock drawing*	1,38	2.029	0.0815	0.081				

For each neuro-cognitive variable, the mean and standard deviation (SD) are reported for healthy controls (HC) and alcoholic patients (AUD), alongside the results of group comparisons with and without statistical control for the effect of age and education (via ANCOVA and two-sample t-tests, respectively). Asterisks denote a non-normal distribution, while bold font denotes a statistically significant effect at p < 0.05, with or without a correction for multiple comparisons based on False Discovery Rate (FDR). ENB: Esame Neuropsicologico Breve 2 (Brief Neuropsychological Examination^44^); DF: degrees of freedom.

This evidence was refined by a multivariate analysis of neuro-cognitive data. Based on the Kaiser-Guttman criterion, considering components with eigenvalues > 1^20^, the initial dataset of 15 ENB2 scores was reduced to 6 components explaining 74.89% of the variance (Supplementary Table S1). These components involve different domains such as visual-constructional abilities, verbal learning, basic-level and high-level executive processes, and language (Table 3). The sixth component, associated with the digit span and cognitive estimation tasks, might reflect the efficiency of processes related to cognitive estimation^21^. A strongly significant group difference was found in the factor score of the third component (F(1,39) = 11.58, p = 0.002). The latter (henceforth “basic-level executive component”) reflects attentional (TMT-A) and working-memory (interference-memory tasks) performance. TMT-A provides the strongest contribution to this component (r = −0.78, p < 0.001), followed by interference-memory-10” (r = 0.71, p < 0.001) and interference-memory-30” (r = 0.69, p < 0.001). Marginal evidence for impaired performance in patients (F(1,39) = 3.74, p = 0.060) was also found in the fourth component (“high-level executive component”), associated with TMT-B, overlapping pictures and abstraction tasks. Therefore, only the basic-level executive component and TMT-A response time (its main contributing variable) were considered in subsequent analyses.

**Table 3:** Principal component analysis of neuro-cognitive data.

A: Principal component	B: Proportion of variance explained (cumulative proportion)	C: ENB2 tests	D: Loading coefficient
#1: Visuo-constructional abilities	15.12%	Praxis abilities	0.916
		Spontaneous drawing	0.791
		Clock drawing	0.619
#2: Verbal learning	14.37% (29.49%)	Delayed recall	0.936
		Immediate recall	0.823
#3: Basic-level executive functions	12.91% (42.4%)	Trail Making test A	−0.779
		Interference memory test 10”	0.711
		Interference memory test 30”	0.686
#4: High-level executive functions	12.01% (54.41%)	Copy drawing	0.839
		Trail Making test B	−0.592
		Overlapping figures	0.496
		Abstract verbal reasoning	0.458
#5: Language	11.49% (65.90%)	Phonemic fluency	0.844
		Token test	−0.830
#6: Estimation-related processes	8.99% (74.89%)	Digit span	0.791
		Cognitive estimation	0.716

The results of a principal component analysis performed on the scores obtained in the Brief neuropsychological examination (ENB2^44^) by alcoholic patients and healthy controls. From left to right, the table reports: the first 6 components (eigenvalue > 1), explaining 74.89% of the total variance of participants’ performance in the 15 ENB2 tests (column A); the relative contribution of each component, in terms of specific and cumulative proportion of variance explained (column B); the single ENB2 tests contributing to each component (column C), and their loading coefficients (column D).

### Grey matter atrophy in alcoholic patients

**Figure 1:**
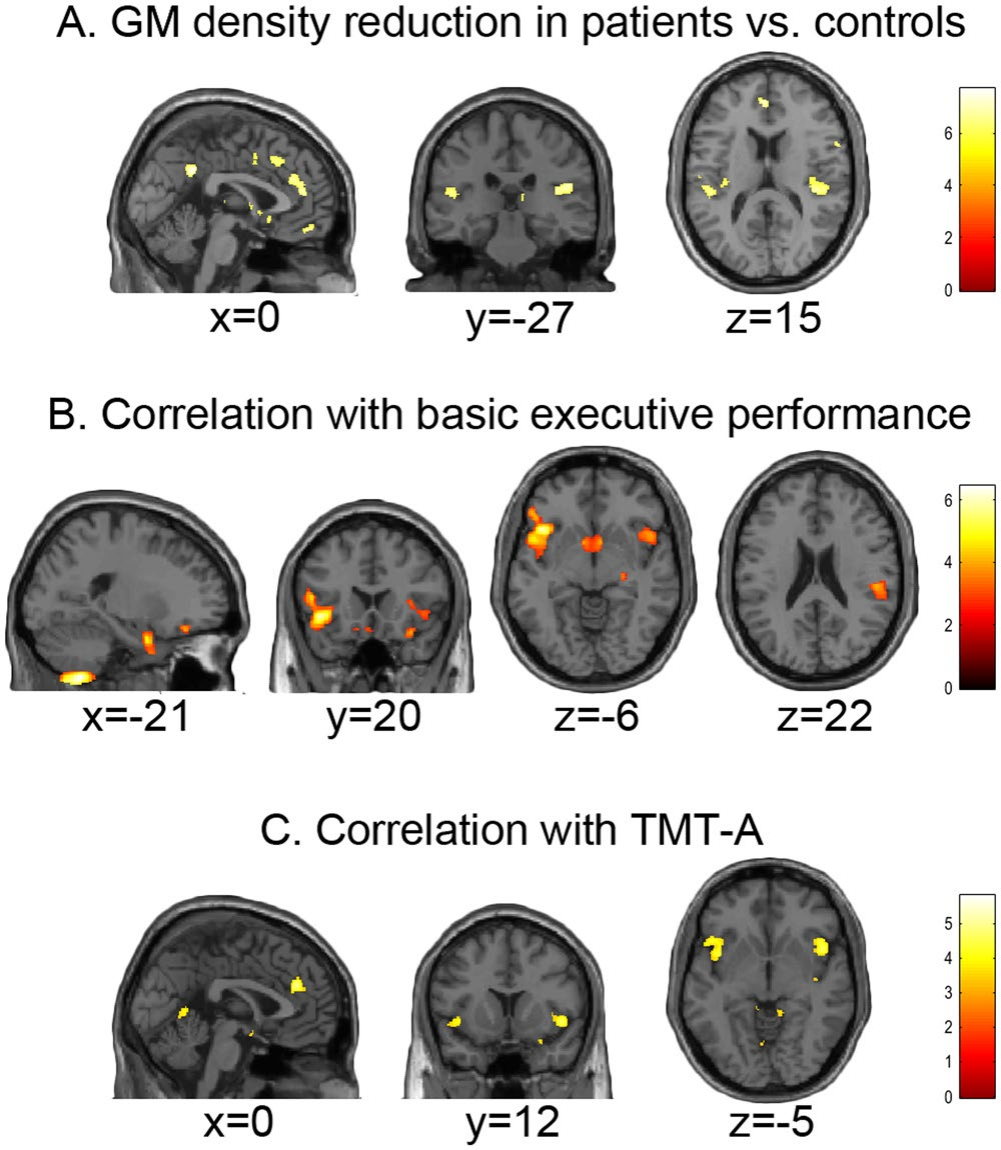
Grey-matter density reduction in AUDs. The brain regions in which grey matter density was (**A**) significantly reduced in AUD patients vs. controls; (**B**) positively correlated with executive performance; (**C**) negatively correlated with TMT-A response time (p < 0.025 corrected for multiple comparisons).

VBM results highlighted a distributed pattern of GM density reduction in alcoholic patients vs. controls (Table 4a; Fig. 1A; Supplementary Table S2). GM atrophy involved both the dorsal (superior medial gyrus and dorsal sector of anterior cingulate cortex (dACC)) and ventral (rectus gyrus) sectors of the medial prefrontal cortex, alongside the pars triangularis of the right inferior frontal gyrus. GM density was significantly reduced, in patients, also in the rolandic operculum and posterior insular cortex, bilaterally but with a right-sided prevalence, as well as in the posterior temporal cortex (superior and middle temporal gyri) bilaterally. Also the left postcentral gyrus and the medial parietal cortex (precuneus and posterior cingulate cortex) displayed a significant GM atrophy, alongside the ventral striatum, caudal thalamus and left amygdala.

**Table 4:** Neuro-structural correlates of executive impairment in AUDs.

H	Brain region	Anatomy toolbox	x	y	z	T	K	TFCE
(a) HC > AUD								
R	Superior medial gyrus		2	24	40	**6.53**	**155**	**7210.86**
L	Anterior cingulate cortex		−2	45	15	**7.37**	**310**	**7538.58**
	Rectus gyrus	Fp2	0	50	−16	**6.62**	**67**	**6835.83**
L	Rolandic operculum		−46	−2	3	**6.02**	**9**	**5624.14**
L	Rolandic operculum	OP2	−36	−24	15	**6.16**	**48**	**6214.18**
R	Anterior insula		36	24	−3	**6.11**	**48**	**5823.82**
R	Rolandic operculum	OP1	50	−27	20	**7.15**	**321**	**6858.31**
R	Posterior insula	Ig2	38	−16	4	**6.21**	**27**	**6408.57**
L	Superior temporal gyrus	OP4	−52	−15	10	**6.64**	**261**	**6431.13**
R	Middle temporal gyrus		54	−18	−9	**7.70**	**148**	**5758.89**
	Posterior cingulate cortex		0	−50	33	**7.59**	**180**	**6980.89**
R	Hippocampus (CA1)		36	−38	−6	**6.73**	**37**	**5589.93**
L	Amygdala		−14	−2	−15	**5.83**	**3**	**6316.35**
R	Ventral striatum		2	2	4	**6.17**	**31**	**7197.22**
L	Thalamus		−10	−32	8	**6.34**	**111**	**7209.67**
R	Thalamus		10	−34	6	**7.34**	**255**	**7771.35**
(b) Correlation between GM density and executive performance								
L	IFG (pars orbitalis)		−42	20	−6	**5.9**	**3755**	**2166.27**
L	Amygdala	LB	−21	2	−27	4.87		**1639.36**
L	Middle orbital gyrus	Fo3	−21	34	−18	3.97	**3495**	**1427.00**
R	Anterior insula		33	12	−18	4.16		**1564.87**
R	Caudate nucleus		8	9	−4	4.08		**1610.74**
R	Medial temporal cortex		12	−10	−15	4.95		**1719.55**
R	Ventral striatum		15	4	−16	4.89		**1756.95**
R	Rolandic operculum		52	−27	22	4.01	**872**	**1386.47**
R	Supramarginal gyrus	PF (IPL)	63	−30	28	3.52		**1348.63**
L	Cerebellum (VIII)	LobuleVIIIa	−24	−57	−58	**6.44**	**1567**	**2231.67**
(c) Correlation between GM density and TMT-A response time								
	dACC		0	38	26	4.94	**279**	**1759.94**
R	Amygdala		21	5	−17	**5.80**	**509**	**1764.37**
R	IFG (pars orbitalis)		29	12	−23	3.51		**1705.77**
L	Insula lobe		−42	9	−5	4.37	**735**	**1763.49**
L	IFG (pars orbitalis)		−48	20	−3	4.28		**1612.84**
L	Temporal pole		−42	18	−15	4.15		**1739.57**
R	Anterior insula		47	12	−6	4.57	**356**	**1742.75**
R	Rolandic operculum	OP4	54	−3	6	4.8	**1173**	**1805.70**
R	Posterior insula	Ig2	39	−15	0	4.27		**1751.76**
L	Rolandic operculum	OP3	−38	−17	18	4.33	**769**	**1749.35**
L	Posterior insula		−33	−21	14	4.32		**1748.74**
R	Fusiform gyrus	FG4	29	−32	−26	4.57	**407**	**1864.69**
R	Lingual gyrus		12	−44	0	5.18	**917**	**1927.88**
L	Calcarine gyrus		−5	−56	3	4.28		**1828.74**
(d) Common effects of AUDs and correlation with executive performance								
L	IFG (pars orbitalis)		−42	20	−3	5.18	**2652**	
L	Anterior insula		−46	9	−6	4.42		
L	Amygdala	LB	−20	3	−27	4.28		
R	Anterior insula		34	18	−18	3.61	**2355**	
R	Middle insula		50	12	−2	4.09		
R	Amygdala		16	−9	−9	4.29		
R	Ventral striatum		16	3	−16	4.5		
R	Rolandic operculum		52	−27	22	4.01	**648**	
(e) Common effects of AUDs and correlation with TMT-A response time								
	dACC		0	38	26	4.94	**1073**	
R	vmPFC/subgenual cortex	s24	2	30	−8	3.02		
R	Medial temporal cortex		10	−9	−16	5.5	**3172**	
R	Ventral striatum		12	3	−16	3.91		
L	Posterior insula		−33	−21	14	4.32	**4594**	
L	Middle insula		−42	9	−4	4.3		
R	Middle insula		46	12	−6	4.57	**1045**	
R	Anterior insula		34	24	3	3.24		
R	Rolandic operculum	OP4	54	−3	6	4.8	**3208**	
R	Posterior insula	Ig2	39	−15	0	4.27		
R	Inferior temporal gyrus		58	−24	−21	4.35	**609**	
R	Lingual gyrus		12	−44	0	5.18	**3173**	
L	Lingual gyrus		−6	−54	3	4.27		
R	Cerebellar Vermis (4/5)		4	−56	4	3.99		
L	Cerebellum (IV-V)		−8	−39	−3	3.65		

From top to bottom, the table reports the regions in which grey matter density was (a) significantly reduced in AUD patients vs. controls; (b) positively correlated with executive performance; c) negatively correlated with TMT-A response time; (d) both significantly reduced in AUD patients vs. controls and positively correlated with executive performance; (e) both significantly reduced in AUD patients vs. controls and negatively correlated with TMT-A response time. See Supplementary Tables S2–S6 for the full list of statistically significant local maxima.

H: hemisphere; TFCE: Threshold-Free-Cluster-Enhancement; HC: healthy controls; AUD: alcoholic patients; L: left; R: right; Fp2: medial frontopolar area 2; OP: parietal operculum; IFG: inferior frontal gyrus; LB: latero-basal amygdala nuclei; Fo3: medial orbital sulcus; IPL: inferior parietal lobule; dACC: dorsal sector of anterior cingulate cortex; FG: fusiform gyrus; K: cluster extent in number of voxels (1 × 1 × 1 mm^3^). Bold font denotes a statistically significant effect at p < 0.025 corrected for multiple comparisons, either at voxel (T), cluster (K) or TFCE levels (note that TFCE statistics are not available for conjunction analysis).

#### Correlation between grey matter density and executive performance

We first assessed a relationship between voxel-wise GM density and the degree of cognitive impairment regardless of group. We found a positive correlation between the scores of the impaired executive component and GM density in fronto-insular and fronto-basal structures, alongside the cerebellum (Table 4b; Fig. 1B; Supplementary Table S3). While the anterior insula was involved bilaterally, in the left hemisphere we observed a wider cluster extending from the lateral orbital gyrus to the posterior insula. Executive performance was also related to GM density in the left amygdala and ventral striatum, in a right-hemispheric posterior insular cluster extending from the rolandic operculum to the superior temporal and supramarginal gyri, as well as in the left cerebellum.

#### Group differences in the correlation between grey matter density and executive performance (interaction analysis)

We found no significant interactive effect of group on the relationship between GM density and executive performance.

#### Correlation between grey matter density and TMT-A response time

Across both groups, TMT-A response time was negatively correlated with GM density in several brain structures (Table 4c; Fig. 1C; Supplementary Table S4), i.e. dACC alongside anterior insula (extending into inferior frontal cortex) and posterior insula (extending into the supramarginal gyrus) bilaterally. An analogous relationship was also observed in the right fusiform gyrus, precuneus, occipital cortex and right amygdala.

#### Group differences in the correlation between grey matter density and TMT-A response time (interaction analysis)

We found no significant interactive effect of group on the relationship between GM density and TMT-A response time.

### Common neuro-structural effects of AUDs and correlation with executive performance

**Figure 2:**
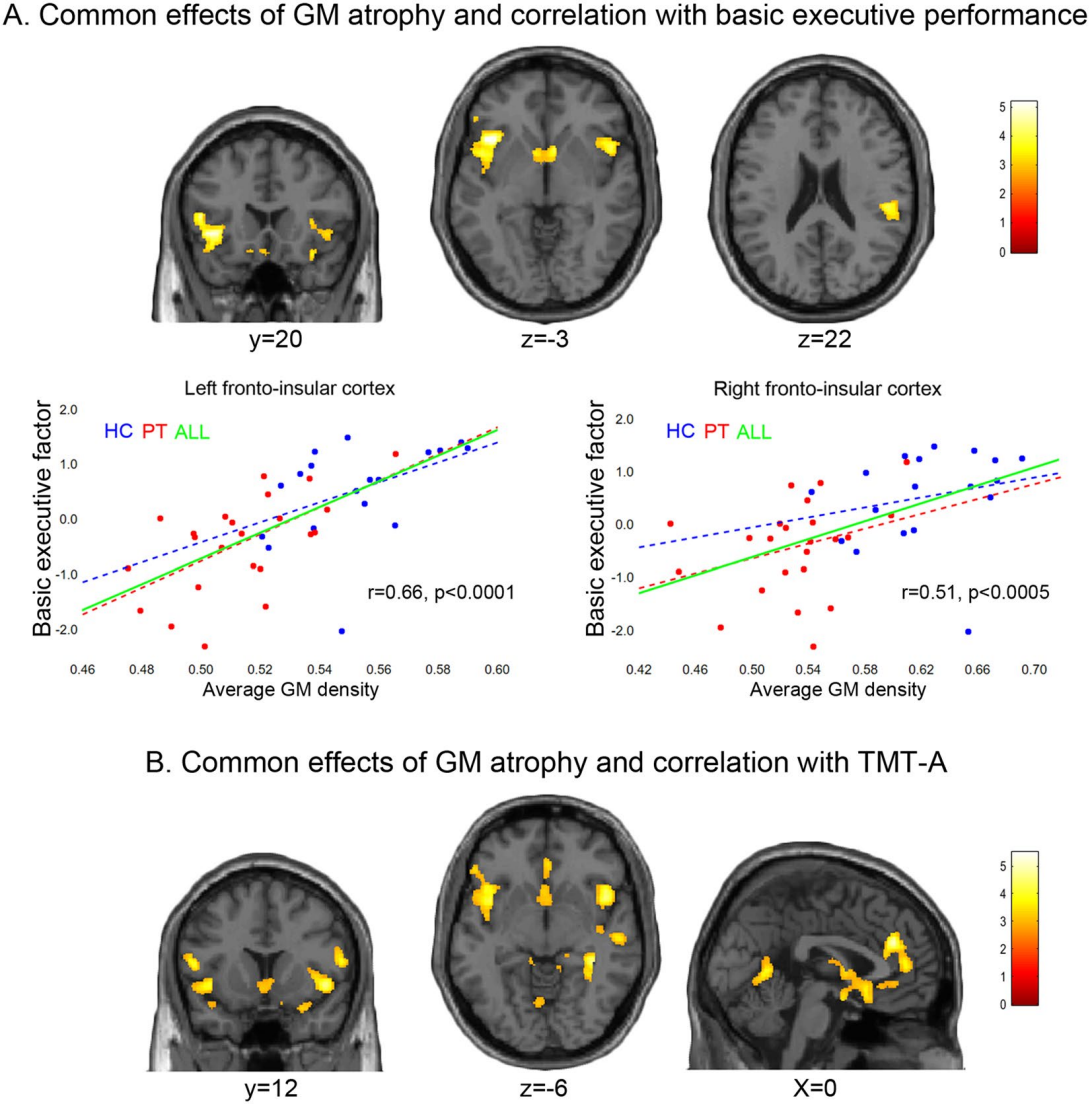
Correlation between grey-matter density and executive performance in AUDs. The brain regions in which grey matter density was (**A**) both significantly reduced in AUD patients vs. controls and positively correlated with executive performance; (**B**) both significantly reduced in AUD patients vs. controls and negatively correlated with TMT-A response time (p < 0.025 corrected for multiple comparisons). The scatterplots in panel (A) additionally depict the relationship between executive performance and average grey matter density in the left and right fronto-insular cortex, either in healthy controls (HC), alcoholic patients (PT) or both.

**Figure 3:**
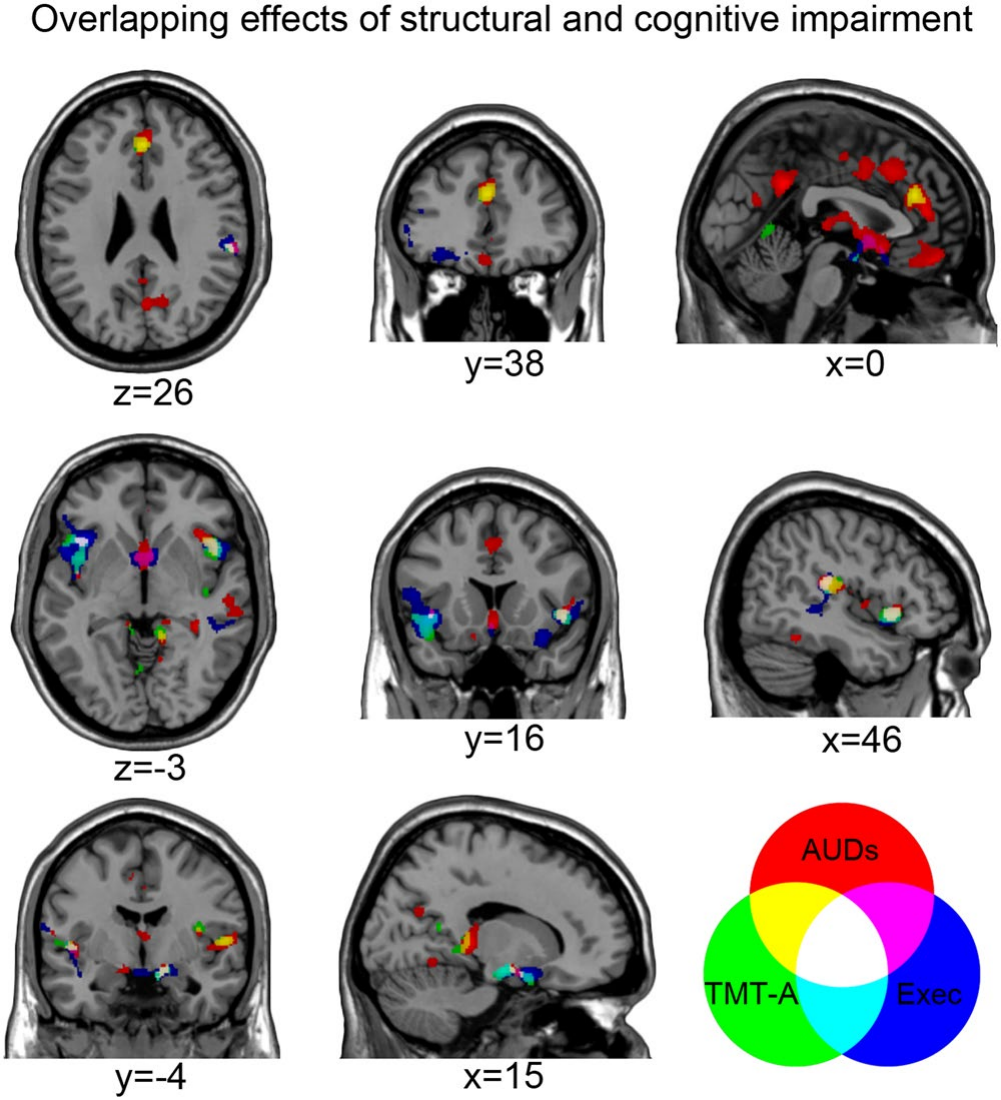
Common neuro-structural effects of AUDs and correlation with executive performance. The brain regions showing specific vs. common effects of AUDs, executive performance or TMT-A response time (p < 0.025 corrected for multiple comparisons).

A conjunction-analysis highlighted the spatial overlap between the voxels associated with the neuro-structural and cognitive impairments observed in alcoholic patients (Figs 2 and 3). This analysis unveiled a clear correspondence between the regions where GM density is a) reduced in patients vs. controls, and b) related to executive performance (Table 4d; Figs 2A and 3; Supplementary Table S5). Such overlap was found bilaterally in a cluster encompassing the anterior-middle insular cortex, as well as in the medial temporal cortex and ventral striatum. We found further common effects in the left inferior frontal cortex (from pars opercularis to pars orbitalis) and right posterior insular cortex (from the rolandic operculum to the superior temporal and supramarginal gyri).

We then extended this approach to the TMT-A task, to investigate a significant overlap between the regions in which GM density was both related to response time and reduced in patients vs. controls (Table 4e; Figs 2B and 3; Supplementary Table S6). Such overlap was found in the dorsal and ventral sectors of the medial prefrontal cortex (dACC and rectus gyrus, respectively), bilateral insula (extending rostrally into the left inferior frontal and caudally into the supramarginal and superior temporal gyri), as well as limbic and basal structures (particularly amygdala and ventral striatum).

### Regions-of-Interest statistical analyses

Homogeneity-of-slopes models confirmed the lack of qualitative group differences in the relationship between GM density and either executive performance or TMT-A response time. The representative scatterplots depicted in Fig. 2A indeed show comparable slopes for the regression of executive performance on GM density in the left (F(1) = 0.28, p = 0.598) and right (F(1) = 0.52, p = 0.474) anterior insular clusters which also displayed a significant atrophy in patients.

When considering all the clusters showing GM atrophy in alcoholic patients (Fig. 1A and Table 4a), a strongly significant multiple regression model (F(1,38) = 11.32, p < 0.0005) showed that 34% of variability in the executive component is explained by average GM density in the dACC (β = 0.94, t(38) = 4.44, p < 0.0001, observed power = 0.99; adjusted r^2^ = 0.34). As expected, constraining such approach to the three clusters in which GM density was also related to executive performance (as highlighted by a conjunction-analysis; Fig. 2A and Table 4d) increased the explained amount of its variance (F(1,38) = 29.43, p < 0.00001; adjusted r^2^ = 0.42). In this case, the only retained predictor was GM density in the left anterior insular cluster.

### Salience network structural integrity and executive impairments

**Figure 4:**
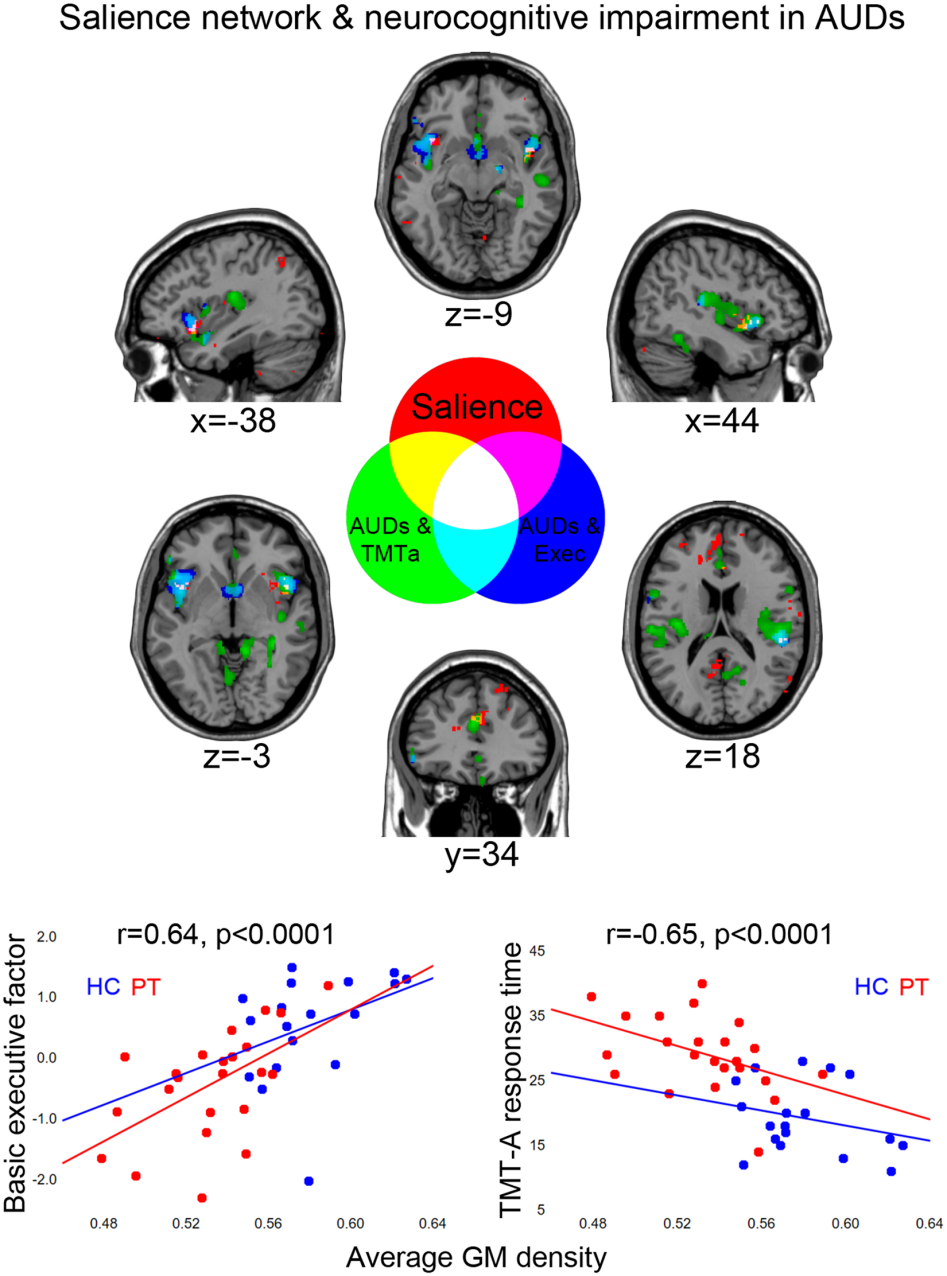
Salience network and executive impairment in AUDs. The overlap between the salience network (red) and the brain regions showing common effects of AUDs and either executive performance (blue) or TMT-A response time (green). The scatterplots depict the significant relationship between average grey matter density in the overlapping voxels (white colour) and either executive performance (left) or TMT-A response time (right).

Based both on our initial hypotheses, and on VBM evidence of structural impairment in the key nodes of the salience network (Figs 1 and 2), we aimed to assess the degree to which such damage accounts for the observed cognitive impairment. To this purpose, we assessed a relationship between executive performance and GM density in the voxels which, besides showing overlapping effects of interest (atrophy in patients, correlation with executive performance and TMT-A response time), were additionally included in the salience network (as highlighted by Neurosynth meta-analytic evidence; see 4.7). Such overlap was found in the anterior insula and frontal operculum bilaterally (left xyz: −38, 18, −10; right xyz: 45 12 −4), alongside right posterior insula (xyz: 50 −30 20) (Fig. 4). Average GM density in this set of regions was significantly correlated with the ENB2 global score (r = 0.33, p = 0.036), and particularly with executive performance (r = 0.64, p < 0.0001) (Fig. 4). The latter relationship appeared to be more strongly driven by TMT-A (r = −0.66, p < 0.0001) than by the other significantly related behavioural measures, i.e. working-memory (interference-memory-10”) and overlapping pictures (both r = 0.38, p = 0.014). Homogeneity-of-slopes models confirmed the lack of significant group differences in the relationship between average GM density in these regions and executive (F(1) = 0.26, p = 0.610) or TMT-A (F(1) = 0.29, p = 0.596) performance.

Overall, GM density in the salience network voxels highlighted by this analysis accounted for 43% and 40% of variability in, respectively, TMT-A and executive performance in the whole sample (p < 0.00001).

## Discussion

We investigated the neural bases of cognitive impairment(s) in AUDs, by coupling a comprehensive neuro-cognitive assessment with neuro-structural VBM evidence of regional GM atrophy.

In patients, a global cognitive impairment was mainly driven by abnormal performance in tasks tapping working-memory (interference memory), visuomotor processing speed and attention (TMT-A), as well as divided attention, switching and mental flexibility (TMT-B; Overlapping pictures). The most defective tasks, i.e. TMT-A and interference-memory, clustered in a strongly impaired basic-level executive domain involving attention and working-memory. Within a global cognitive impairment^22^, our data thus confirm the prominence of an executive disorder in AUDs^23^. It is still debated, however, whether this deficit reflects a specific susceptibility of frontal regions (“frontal lobe” hypothesis^6^), or a diffuse pattern involving other cortical or subcortical structures (“diffuse brain hypothesis”^7^). We used VBM to distinguish between these hypotheses, by providing a detailed characterization of the neuro-structural bases of cognitive impairment in AUDs.

In line with previous data^9^, VBM results highlighted a diffuse pattern of GM decrease, in patients, along the subcortical (ventral striatum, thalamus, hippocampus and amygdala,) and cortical (ventromedial and posterior dorsomedial frontal cortex) components of the meso-cortico-limbic pathway (Fig. 1A). Based on functional magnetic resonance imaging (*f*MRI) evidence for its role in adaptive behavioural learning^24,25^, an impairment of this pathway has been suggested to underpin the development and maintenance of addiction, via negative reinforcement^26^. GM atrophy was also found in the dACC and insular cortex^27^, i.e. the main nodes of the so-called “salience network”^10^. This evidence of widespread brain damage fits with the notion that the cognitive and behavioural alterations observed in substance-use disorders, including AUDs, might reflect functional imbalances within a cortico-striato-thalamo-cortical regulatory loop underlying executive control and self-regulation^10^.

This circuit underpins another crucial component of behavioural learning^28^, i.e. regulating the switch between rest and effortful cognitive activity based on the salience of external stimuli with respect to behavioural goals. This mechanism is driven by the salience network, involving both cortical (dACC and anterior insula) and subcortical (basal ganglia, thalamus and amygdala) nodes of the loop^10,12^. Their functional interaction is central to detect salient (i.e., motivationally relevant) stimuli, and to enable them to modulate cognition and behaviour via executive processes of response inhibition and selection associated with the lateral prefrontal cortex^10^. This neural mechanism facilitates the access to attention and working-memory resources, by activating the dorsal attentional and fronto-parietal executive control networks, when relevant stimuli are detected^12^. The vicious circle linking executive and behavioural impairments might then be further reinforced, in AUDs, by abnormal processing of salient stimuli, due to the structural impairment of the key nodes of the salience network. To test this hypothesis, we assessed the spatial overlap between the regions displaying atrophy in patients and those in which GM density relates to impaired cognitive performance.

We first assessed the relationship between GM density and performance in the executive domain showing the greatest impairment in alcoholic patients. The lack of significant task-by-group interactions suggests that no qualitative difference exists, between patients and controls, in this relationship. Instead, correlational analyses highlighted evidence of *quantitative* group differences in this respect (Figs 1B and 2A): in the whole sample, executive performance was positively correlated with GM density in different clusters encompassing fronto-insular and medial temporal cortex bilaterally, alongside right posterior insular cortex, left cerebellum and ventral striatum. While showing a clear segregation between patients and controls, the scatterplots displayed in Fig. 2A indeed highlight, in the two groups, comparable slopes in the relationship between executive performance and GM density in the left and right anterior insula.

In line with behavioural evidence, TMT-A response time provided the strongest contribution in explaining the relationship between brain atrophy and executive dysfunction in AUDs. Moreover, the lack of a significant task-by-group interaction confirmed that such relationship reflects quantitative, rather than qualitative, differences along a continuum from normal to impaired conditions. In the case of TMT-A this relationship involves a general slowing of performance associated with grey matter loss in regions largely overlapping those previously described for executive performance, with the additional contribution of the dACC (Figs 1C–4). The involvement of this structure supports the relationship between executive performance, particularly in the TMT-A, and GM density in the salience network^10^. Figure 4 (white colour) indeed shows the overlap between a meta-analytic map of this network and the bilateral anterior and right posterior insular clusters showing both atrophy in alcoholic patients and a significant correlation between GM density and executive performance. In the whole sample, average GM density in these voxels accounted for almost half of variability in TMT-A response time (adjusted r^2^ = 43%) and executive performance (adjusted r^2^ = 40%).

Overall, these data support an interpretation of AUD patients’ executive impairment in terms of defective access to attention and working-memory resources, due to the structural damage of a salience-detection neural mechanism in charge of activating the executive network^12^. In particular, the insular cortex is considered to link perceptual, cognitive and autonomic information, by relaying salience signals related to body states, generated by viscero-autonomic sensors and transmitted by thalamic nuclei^29^. An interoceptive awareness of salient stimuli involves the posterior insular sector of the network, which we also found to be both related to executive performance in our sample, and structurally impaired in patients. These data might thus reflect a domain-general role of the insular salience node in enhancing access to computational resources. Instead, the specific association between TMT-A response time and GM density in the dACC sector of the salience network (Fig. 4, yellow colour) may reflect the prominent role of this region in conflict monitoring and response selection^30^.

Within a diffuse pattern of GM atrophy, our data thus highlight an impairment of the salience network as a specific neuro-structural correlate of executive dysfunction in AUDs, likely related to previous evidence of defective functional activation of the executive network^31^. The present evidence of a more severe impairment of the insular and dACC nodes of the salience network, compared with the lateral prefrontal nodes of the executive network, fits with a recent meta-analysis of previous VBM studies on AUDs^9^. The prominent damage of insular and anterior cingulate cortex likely reflects the susceptibility of so-called von Economo neurons, localized in these regions^32^, to the neurotoxic effects of alcohol^27^. Importantly, there are limitations to interpreting the functional significance of neuro-structural data. However, the present results complement previous evidence at the functional level, i.e. abnormal connectivity between the insular and dACC nodes of the salience network highlighted by studies based both on *f*MRI^33^ and arterial spin labelling^34^.

A limitation of this study is represented by the lack of in-depth measures of executive functioning and cognitive control, such as response inhibition; in the trade-off between comprehensiveness and specificity, however, opting for a broad neuropsychological assessment allowed us to highlight the selective impairment of an executive domain transcending specific attentional and working-memory tasks. This novel evidence contributes to a lively debate between two opposite views rooted in the focal vs. global nature of the cognitive and neural dysfunctions in AUDs, and that our data seem to reconcile. The cognitive impairment in different tasks, associated with functional alterations in multiple brain structures, might indeed reflect the impairment of a specific executive domain, due to the structural damage of a well-established salience network activating the executive control network when relevant stimuli are detected. Another limitation is represented by a relatively small sample; by focusing on few cognitive variables showing the strongest impairment in patients, however, we identified a strongly significant relationship between its severity and the extent of GM atrophy (Fig. 4). Finally, we report only cross-sectional evidence which will require further support from future longitudinal studies.

Identifying a specific neural substrate of executive impairment in AUDs has several implications. First, we observed quantitative, rather than qualitative, group differences in the relationship between GM density and domain- or task-specific executive performance. This evidence might seem to challenge the notion of adaptive neural mechanisms supporting cognitive performance via compensatory brain regions^35^. Such neuroadaptation, however, might become detectable at the structural level only after longer abstinence periods. Second, the salience-detection mechanism discussed above might also influence decision-making, and thus abstinence vs. relapse, by underpinning the interplay between a “reflexive” limbic system driving automatic behaviours and a “reflective” fronto-striatal network activating executive control^23^. A defective switching mechanism between these systems may thus bias decision-making processes towards bottom-up impulsive signals, at the expenses of top-down goal-driven attentional resources required to exert behavioural control over alcohol search and consumption^36,37^. This hypothesis fits with previous morphometric evidence showing that AUD patients’ increased impulsivity correlates with the degree of grey matter atrophy in the anterior insular and cingulate nodes of the salience network^11^.

The present results highlight several directions for future research. Growing evidence shows that long-term abstinence can reverse both behavioural^5,38^ and neuro-structural^13,14,39^ alterations. Moreover, preliminary evidence highlights positive effects of cognitive remediation^40^ and neurostimulation^41^ on patients’ cognitive performance and craving. However, treatment efficacy depends on the integrity of executive functions^42^, and the presence of cognitive impairment requires adapting management strategies based on individual profiles^43^. Therefore, both the design of treatment protocols, and the assessment of their effectiveness, require sensitive benchmark metrics of executive functioning. Moreover, the efficacy of neurostimulation protocols depends on the choice of the target area, which in turn will reflect previous evidence of a significant relationship between its structural or functional properties and cognitive performance. By showing a restricted set of tasks which are particularly sensitive to AUD patients’ executive impairment, and its neural correlates, our results might thus help tailor remediation or neurostimulation treatment protocols to target specific brain networks and their associated cognitive functions.

## Methods

See Supplementary methods for additional details.

### Participants

Twenty-three alcoholic patients (9 females; mean age: 45.69 years ± 7.82) and 18 healthy control subjects (8 females; mean age: 44.83 years ± 8.86) participated in the study. Patients were interviewed to determine their drinking history, including the amount, type and lifetime duration of alcohol usage. Alcohol consumption was calculated as the average number of standard units of alcohol (UA) per day (one UA: 330 ml beer, 125 ml wine, or 40 ml hard liquor, corresponding to 12 g of ethanol) (see Table 1 for demographic, as well as nicotine and alcohol use, variables).

Inclusion criteria for patients were: (1) age between 20 and 60 years; (2) a diagnosis of alcohol dependence according to DSM-V criteria. Exclusion criteria for both groups were: (1) presence/history of neurological/psychiatric disorders other than AUDs, or any comorbid disorder except for nicotine dependence; (2) family history of neurological/psychiatric disorders; (3) current use of any psychotropic substance/medication; (4) past brain injury or loss of consciousness; (5) major medical disorders (e.g. kidney or liver diseases, sever diabetes and/or malnutrition); (6) inability to undergo the neuropsychological assessment; (7) contraindications to magnetic resonance imaging (MRI). Controls were excluded in case of presence/history of alcohol abuse. Patients joined the protocol after being detoxified for at least 10 days, via medically supported standard treatments. However, they had ceased benzodiazepine treatment at least 8 days before scanning. Controls were at least abstinent 10 days before scanning. All participants provided written informed consent to the experimental procedure, which was approved by the Ethical Committee of ICS Maugeri (Pavia, Italy). The investigation was conducted in accordance with the latest version of the Declaration of Helsinki.

### Neuro-cognitive assessment

All participants underwent a neuro-cognitive evaluation based on the Brief neuropsychological examination^44^, a well-validated battery for the Italian population including tasks for different cognitive domains: attention (trail making (TMT-A and TMT-B)), memory (digit span, immediate and delayed prose memory), working-memory (10- and 30-seconds interference-memory), executive functions (TMT-B, cognitive estimation, abstract reasoning, phonemic fluency, clock drawing and overlapping pictures), as well as perceptive and praxis skills (praxis abilities, spontaneous drawing and copy drawing task). The battery results in a score for every task, alongside an overall score of global cognitive status.

### Statistical analysis of neuro-cognitive data

For each task, we checked the normality of the score distribution across the whole sample. Based on this assessment, we then examined age and group effects by means of parametric or non-parametric two-sample and correlation tests. For the tasks showing a significant effect of age or education, we run an Analysis of covariance (ANCOVA) to assess group differences on cognitive performance after removing their effect. We applied a primary statistical threshold of p < 0.05, one-tailed due to a priori hypotheses of cognitive impairment in AUDs^7,45^, and then performed a correction for multiple comparisons based on FDR.

We investigated superordinate cognitive domains, transcending specific tasks, in which performance was impaired in patients. After assessing the suitability of the correlation matrix (Keiser-Meyer-Olkin Measure of Sampling Adequacy = 0.61; Bartlett’s test of sphericity <0.001; Supplementary Table S7) we performed a principal component analysis on the 15 ENB2 raw scores. Due to the ambiguity of the scree plot (Supplementary Fig. 1), we used the Kaiser-Guttman criterion to determine the number of components to be retained (i.e., components with eigenvalue >1). An orthogonal rotation (Varimax) was used to facilitate the interpretation of components^20^ (Supplementary Tables S8,S9). To investigate group differences in cognitive performance, we used an ANOVA (with Bonferroni correction for multiple comparisons) on the resulting factor scores for each subject/component.

### MRI data acquisition

We used a 3 Tesla General Electrics Discovery scanner to collect a high-resolution 3D T1-weighted IR-prepared FSPGR (BRAVO) brain scan acquired along the AC-PC plane (152 slices, FOV = 24 cm, reconstruction matrix = 256 × 256, slice thickness = 1 mm). A T2-weighted image was also collected for diagnostic purposes.

### VBM data pre-processing and whole-brain statistical analysis

We performed image pre-processing and statistical analyses using SPM12 (http://www.fil.ion.ucl.ac.uk/spm) and the CAT12 toolbox (http://www.neuro.uni-jena.de/cat/). The pre-processing included a correction for bias-field inhomogeneities, spatial normalization using the DARTEL algorithm^46^, segmentation into GM, white matter (WM) and cerebrospinal fluid (CSF)^47^, and smoothing with an 8 mm gaussian kernel.

Statistical analyses included: (a) a two-sample t-test, to assess a decrease of GM density in alcoholic patients vs. controls; (b) multiple regressions, to assess a relationship between GM density and performance in the domain/task displaying the greatest impairment in patients; and (c) full factorial models (two-sample t-test plus a behavioural covariate), to assess group differences in the relationship between GM density and performance (i.e., a significantly different regression slope in patients vs. controls). For (b) and (c), in separate analyses we modelled either the factor score of the basic-level executive component, or response-time of the TMT-A task to examine its contribution to the component. Multiple regressions and full factorial models highlighted, respectively, quantitative or qualitative group differences in the relationship between GM density and domain/task performance. We modelled age to remove its potentially confounding effect, and applied an internal GM threshold of 0.15 to prevent artefacts on the GM-WM border due to voxel misclassification. We used conjunction-null analyses^48^ to assess the predicted anatomical overlap between the regions in which GM density was both reduced in patients vs. controls, and related to executive or TMT-A performance.

Since the above analyses involved two behavioural measures, we adjusted our primary statistical threshold to p < 0.025 corrected for multiple comparisons with FDR (as implemented in SPM12) at the voxel or cluster level. We applied threshold-free cluster enhancement (TFCE^49^) with 5000 permutations per contrast and correction for multiple comparisons. This approach has been shown to increase the sensitivity of VBM findings^50^.

### Regions-of-Interest statistical analyses

We investigated whether, and to what extent, the pattern of cognitive impairment observed in alcoholic patients is explained by the degree of regional GM atrophy. We used the SPM toolbox Marsbar (http://marsbar.sourceforge.net/) to create binary masks of the clusters displaying the effects reported above, i.e. a) GM atrophy in patients vs. controls; b) common effect of GM atrophy in patients *and* correlation with basic-level executive performance. Using the SPM toolbox REX (http://web.mit.edu/swg/software.htm), the average GM density in these regions was extracted for each subject, and entered in offline analyses. Namely, we used average GM density in the observed clusters as simultaneous predictors of a multiple regression model, to assess their global and relative efficacy for predicting executive performance.

### Meta-analytic evidence of an overlap with the salience network

We used the Neurosynth toolbox (http://neurosynth.org) to produce a meta-analytic map of the salience network (see Supplementary Methods), and then examine its spatial overlap with the regions displaying common effects of interest (atrophy in patients, correlation with executive performance and TMT-A response time).

We used the Marsbar toolbox, as described above, to create spatial maps corresponding to the conjunction of our effects of interest, i.e. inclusion in the salience network alongside significant GM atrophy in AUDs, correlation with executive performance and with TMT-A response time. Then, to evaluate the extent to which the morphometric properties of the resulting regions account for cognitive performance, we replicated the procedure described above to extract average GM density from the commonly involved voxels, for offline multiple regressions.

## Electronic supplementary material

Supplementary information

## Data Availability

The datasets generated and/or analysed during the current study are available from the corresponding author on reasonable request.
