# Supplementary material for: Salience network structural integrity predicts executive impairment in alcohol use disorders
Source: Sci Rep. 2018 Sep 27;8:14481. doi: 10.1038/s41598-018-32828-x (PMC6160480; doi:10.1038/s41598-018-32828-x)
Supplement: Supplementary file 1 — Supplementary information [file 41598_2018_32828_MOESM1_ESM.pdf]

Salience network structural integrity predicts executive impairment in alcohol use disorders

Caterina Galandra[1,2,3], Gianpaolo Basso[3,4], Marina Manera[5] , Chiara Crespi [1,2], Ines Giorgi[5], Giovanni Vittadini[6], Paolo Poggi[7], Nicola Canessa[1,2,*]

1 Scuola universitaria superiore IUSS, Pavia, 27100, Italy
2 Cognitive neuroscience laboratory, ICS Maugeri, Pavia, 27100, Italy
3 LabNIT, ICS Maugeri, Pavia, 27100, Italy
4 University of Milano-Bicocca, Milan, 20126, Italy
5 Clinical psychology unit, ICS Maugeri, Pavia, 27100, Italy
6 Functional rehabilitation unit, ICS Maugeri, Pavia, 27100, Italy
7 Radiology unit, ICS Maugeri, Pavia, 27100, Italy

* Corresponding author: nicola.canessa@iusspavia.it; Scuola universitaria superiore IUSS, Pavia, 27100, Italy; Cognitive neuroscience laboratory, ICS Maugeri, Pavia, 27100, Italy. Phone: +39-0382-375845; Fax: +39-0382-375899.

SUPPLEMENTARY METHODS

Participants

Twenty-three adult alcoholic patients (9 females; mean age: 45.69 years±7.82; range: 29-58; mean education: 10.11 years±2.78) and 18 healthy control subjects (7 females; mean age: 44.83 years±8.86; range: 27-57; mean education: 10.00 years±2.62) participated in the study. A chi-square test confirmed that the distribution of males and females was not significantly different across patients and controls (p=0.9874). Patients were enrolled from the Functional rehabilitation Unit of ICS Maugeri-Pavia (Italy), and healthy controls were recruited via local advertisement. There was no significant group difference concerning age, education or nicotine consumption (Table 1). Patients were interviewed to determine their drinking history, including the amount, type and lifetime duration of alcohol usage. Alcohol consumption was calculated as the average number of standard units of alcohol (UA) per day (one UA: 330 ml beer, 125 ml wine, or 40 ml hard liquor, corresponding to 12 g of ethanol) (see Table 1 for alcohol use variables). Average disease duration in patients ranged from 1 to 26 years (mean: 10.8 years±7.21).

Inclusion criteria for alcoholic patients were: 1) age between 20 and 60 years; 2) a diagnosis of alcohol dependence according to DSM-V criteria. Exclusion criteria for both alcoholic patients and control subjects were: 1) presence or history of neurological or psychiatric disorders other than AUDs, or any comorbid disorder except for nicotine dependence; 2) family history of neurological or psychiatric disorders; 3) current use of any psychotropic substance or medication; 4) past brain injury or loss of consciousness; 5) major medical disorders (e.g. kidney or liver diseases, sever diabetes and/or malnutrition); 6) inability to undergo the neuropsychological assessment; 7) contraindications to magnetic resonance imaging (MRI). Healthy controls were also excluded in case of presence or history of alcohol abuse. Patients joined the experimental protocol after being detoxified for at least 10 days, via medically supported standard treatments. However, they had ceased benzodiazepine treatment at least 8 days before scanning. Healthy participants were at least abstinent 10 days before scanning. All participants provided written informed consent to the experimental procedure, which was approved by the local Ethical Committee.

Neuro-cognitive assessment

All patients and healthy controls underwent a neuro-cognitive evaluation based on the Brief neuropsychological examination (Esame Neuropsicologico Breve 2 (ENB2); [1]), a well-validated battery for the Italian population including tests for different cognitive domains: attention (trail making (TMT-A and

TMT-B)), memory (digit span, immediate and delayed prose memory), working-memory (10- and 30-seconds interference-memory), executive functions (TMT-B, cognitive estimation, abstract reasoning, phonemic fluency, clock drawing, and overlapping pictures), as well as perceptive and praxis skills (praxis abilities, spontaneous drawing and copy drawing tests). The TMT-B test is listed twice because it involves two domains, i.e. attention and executive functioning (particularly working-memory and switching). The battery results in a score for every task, alongside an overall score of global cognitive status.

Analysis of neuro-cognitive data

For each test, we first checked the normality of the score distribution across the whole sample. Based on the results of this assessment, we then examined age and group effects by means of parametric (Pearson's correlation index and two-sample t-test, respectively) or non-parametric (Spearman's correlation index or Mann-Whitney t-test, respectively) statistical tests. For the tests showing a significant effect of age or education, we additionally run an Analysis of covariance (ANCOVA) to assess group differences on cognitive performance after removing their effect. We applied a primary statistical threshold of $p<0.05$, one-tailed due to a priori hypotheses of cognitive impairment in AUDs [2-3] (see Introduction), and then performed a correction for multiple comparisons based on FDR.

We investigated superordinate cognitive domains, transcending specific tasks, in which performance was impaired in patients. After assessing the suitability of the correlation matrix (Keiser-Meyer-Olkin Measure of Sampling Adequacy = 0.61; Bartlett's test of sphericity < 0.001; Supplementary Table S7) we performed a principal component analysis on the 15 ENB2 raw scores. Due to the ambiguity of the scree plot (Supplementary Figure 1), we determined the number of components to be retained by applying the Kaiser-Guttman criterion (i.e., components with eigenvalue>1) (Supplementary Tables S8-S9). An orthogonal rotation (Varimax) was used to facilitate the interpretation of components [4-5]. To investigate group differences in cognitive performance, we used an ANOVA (with Bonferroni correction for multiple comparisons) on the resulting factor scores for each subject/component.

MRI data acquisition

We used a 3 Tesla General Electrics Discovery scanner (GE Healthcare, Milwaukee, WI), equipped with an 8-channels head coil, to collect a high-resolution 3D T1-weighted IR-prepared FSPGR (BRAVO) brain scan (TR=8.2 ms, TE=3.2 ms, FA=12°, TI=450 ms, NEX=1, FOV=24 cm, reconstruction matrix=256 x 256, slice thickness=1 mm, 152 slices) acquired along a semi-axial orientation parallel to the AC-PC plane, covering the whole brain from the occipital foramen to the region above the vertex. A T2-weighted image was also collected, and interpreted by an experienced neuroradiologist for diagnostic purposes.

VBM data preprocessing and whole-brain statistical analysis

We performed image preprocessing and statistical analyses using SPM12 (http://www.fil.ion.ucl.ac.uk/spm) and the CAT12 toolbox (www.neuro.uni-jena.de/cat/) running on Matlab v9.0 (Mathworks, Inc., Sherborn, MA).

All the T1-weighted images were first inspected for motion artefacts and gross anatomical abnormalities, and the origin of the plane was set on the anterior commissure. By means of the CAT12 toolbox, images were then corrected for bias-field inhomogeneities, spatially normalized using the DARTEL algorithm [6] and segmented into GM, white matter (WM) and cerebrospinal fluid (CSF) [7]. Compared with the SPM12 pipeline, CAT12 improves the segmentation process via the estimation of partial volume effects [8], adaptive maximum a posteriori estimations [9] and a hidden Markov Random Field model [10]. We did not apply the jacobian modulation of segmented GM images, which corrects for volume change during spatial normalization, since this procedure has been shown to decrease the sensitivity to morphometric abnormalities [11]. Our results thus involve GM density, i.e. GM volume relative to WM and CSF volume. Finally, we applied a smoothing kernel of 8 mm (FWHM) to the normalized segmented GM images.

After preprocessing, all images underwent both a visual check for artefacts and an automated check of sample homogeneity based on the degree of correlation across images. Both procedures led to retain all images, since no clear outlier was identified. In addition, we found no group difference in terms of two quality parameters describing the image properties before CAT12 processing, i.e. "noise contrast ratio" ($p=0.1919$) and "weighted average image quality rating" (IQR; $p=0.1960$).

We performed whole-brain statistical analyses by means of the General Linear Model approach, as implemented in SPM12. Namely, we modeled: a) a two-sample t-test, to assess a significant decrease of GM density in alcoholic patients vs. controls; b) multiple regressions, to assess a significant relationship between GM density and performance in the domain/task displaying the greatest impairment in patients; c) full factorial models (two-sample t-test plus a behavioral covariate) to assess significant group differences in the relationship between GM density and performance (i.e. a significantly different regression slope in in patients vs. controls). For both b) and c), in separate analyses we modelled either the factor score of the basic-level executive component, or response-time of the TMT-A task to examine its contribution to the component. Multiple regressions and full factorial models highlighted, respectively, quantitative or qualitative group differences in the relationship between GM density and domain/task performance. We modeled age to remove its potentially confounding effect. We modelled age to remove its potentially confounding effect, and applied an internal GM threshold of 0.15 to prevent artefacts on the GM-WM border due to voxel misclassification. We used conjunction-null analyses [12] to assess the predicted anatomical overlap between the regions in which GM density was both reduced in patients vs. controls, and related to executive or TMT-A performance.

Since the above analyses involved two behavioural measures, we adjusted our primary statistical threshold to $p<0.025$ corrected for multiple comparisons with FDR (as implemented in SPM12) at the voxel or cluster level. We applied threshold-free cluster enhancement (TFCE; [13]) with 5000 permutations per contrast and correction for multiple comparisons. This approach has been shown to increase the sensitivity of VBM findings [14].

Region-of-Interest analyses

We then aimed to investigate whether, and to what extent, the pattern of cognitive impairment observed in alcoholic patients is explained by the degree of regional GM atrophy. To this purpose, we first used the SPM toolbox Marsbar (http://marsbar.sourceforge.net/) to create binary masks of the clusters displaying the different main effects reported above, i.e. a) GM atrophy in patients vs. controls; b) common effect of GM atrophy *and* correlation with basic-level executive performance. Using the SPM toolbox REX (http://web.mit.edu/swg/software.htm), the average GM density in these regions was extracted for each subject, and entered in offline analyses. Namely, we used average GM density in the observed clusters as simultaneous predictors of a multiple regression model, to assess their global and relative efficacy for predicting executive performance.

Overlap with the salience attentional network

Based on the results of the above analyses, we examined the spatial overlap between the regions displaying common effects of interest (i.e. atrophy in patients, correlation with executive and TMT-A performance) and those included in the salience network [15] in charge of switching between the default mode and executive control networks [16-17]. We grounded this investigation in the meta-analytic approach implemented by the Neurosynth toolbox (http://neurosynth.org), which allows to quantify the specificity and consistency of brain activity in association with a given cognitive process [18].

The current Neurosynth database includes coordinates from 11406 published studies, associated with over 413429 reported activations, automatically extracted from the available literature regardless of specific brain regions or processes of interest. This information is used to generate meta-analytic maps for several thousand psychological terms [19], i.e. 3107 as of January 2018, which can be accessed via a search interface displaying all studies containing a specified keyword. This procedure implements two types of brain-process inferences, i.e. forward (probability of observing activity in a region given knowledge of the psychological process) and reverse (probability of a psychological process being present given knowledge of activation in a specific brain region) [20]. These two approaches highlight, respectively, how consistently a task/state activates a region, and how specific its activation is to the task/state, with the latter being closer to the notion of decoding mental states from brain activity [21].

We used the Neurosynth interface to produce reverse inference maps associated with the “salience network”. The resulting spatial map, based on 60 previous studies and 2327 activations, was first used to assess the spatial overlap between this network and the regions underlying the predicted effects of interest in AUDs. To this purpose, we used the Marsbar toolbox, as described above, to create spatial maps

corresponding to the conjunction of all our four effects of interest, i.e. inclusion in the salience network alongside significant GM atrophy in AUDs, correlation with executive performance and with TMT-A response time. Then, to evaluate the extent to which the morphometric properties of the resulting brain regions accounts for cognitive performance, we replicated the procedure described above to extract average GM density from the commonly involved voxels, for subsequent offline multiple regression analyses.

REFERENCES

1. Mondini, S., Mapelli, D., Vestri, A., Arcara, G. & Bisiacchi, P. *Esame neuropsicologico breve. Una batteria di test per lo screening neuropsicologico.* (Raffaello Cortina, 2011);
2. Le Berre, A.-P., Fama, R. & Sullivan, E. V. Executive Functions, Memory, and Social Cognitive Deficits and Recovery in Chronic Alcoholism: A Critical Review to Inform Future Research. *Alcohol. Clin. Exp. Res.* **41,** 1432–1443 (2017);
3. Stavro, K., Pelletier, J. & Potvin, S. Widespread and sustained cognitive deficits in alcoholism: A meta-analysis. *Addict. Biol.* **18,** 203–213 (2013);
4. Abdi, H. & Williams, L. J. Principal component analysis. *Wiley Interdisciplinary Reviews: Computational Statistics* **2,** 433–459 (2010);
5. Jolliffe, I. Principal Component Analysis. *Int. Encycl. Stat. Sci.* 1094–1096 (2011);
6. Ashburner, J. A fast diffeomorphic image registration algorithm. *Neuroimage* **38,** 95–113 (2007);
7. Ashburner, J. & Friston, K. J. Unified segmentation. *Neuroimage* **26,** 839–851 (2005);
8. Tohka, J., Zijdenbos, A. & Evans, A. Fast and robust parameter estimation for statistical partial volume models in brain MRI. *Neuroimage* **23,** 84–97 (2004);
9. Rajapakse, J. C., Giedd, J. N. & Rapoport, J. L. Statistical approach to segmentation of single-channel cerebral MR images. *IEEE Trans. Med. Imaging* **16,** 176–186 (1997);
10. Cuadra, M. B., Cammoun, L., Butz, T., Cuisenaire, O. & Thiran, J.-P. Comparison and validation of tissue modelization and statistical classification methods in T1-weighted MR brain images. *IEEE Trans. Med. Imaging* **24,** 1548–1565 (2005);
11. Radua, J., Canales-Rodríguez, E. J., Pomarol-Clotet, E. & Salvador, R. Validity of modulation and optimal settings for advanced voxel-based morphometry. *Neuroimage* **86,** 81–90 (2014);
12. Nichols, T., Brett, M., Andersson, J., Wager, T. & Poline, J.-B. Valid conjunction inference with the minimum statistic. *Neuroimage* **25,** 653–660 (2005);
13. Smith, S. M. & Nichols, T. E. Threshold-free cluster enhancement: Addressing problems of smoothing, threshold dependence and localisation in cluster inference. *Neuroimage* **44,** 83–98 (2009);
14. Radua, J., Canales-Rodríguez, E. J., Pomarol-Clotet, E. & Salvador, R. Validity of modulation and optimal settings for advanced voxel-based morphometry. *Neuroimage* **86,** 81–90 (2014).
15. Peters, S. K., Dunlop, K. & Downar, J. Cortico-Striatal-Thalamic Loop Circuits of the Salience Network: A Central Pathway in Psychiatric Disease and Treatment. *Front. Syst. Neurosci.* **10,** 1–23 (2016);
16. Menon, V. & Uddin, L. Q. Saliency, switching, attention and control: a network model of insula function. *Brain Struct. Funct.* **214,** 655–667 (2010);
17. Sridharan, D., Levitin, D. J. & Menon, V. A critical role for the right fronto-insular cortex in switching between central-executive and default-mode networks. *Proc. Natl. Acad. Sci.* **105,** 12569–12574 (2008);
18. Yarkoni, T., Poldrack, R. A., Nichols, T. E., Van Essen, D. C. & Wager, T. D. Large-scale automated synthesis of human functional neuroimaging data. *Nat. Methods* **8,** 665–70 (2011);
19. Poldrack, R. A. *et al.* The Cognitive Atlas: Toward a Knowledge Foundation for Cognitive Neuroscience. *Front. Neuroinform.* **5,** 17 (2011);
20. Poldrack, R. Can cognitive processes be inferred from neuroimaging data? *Trends Cogn. Sci.* **10,** 59–63 (2006);
21. Chang, L. J., Yarkoni, T., Khaw, M. W. & Sanfey, A. G. Decoding the role of the insula in human cognition: Functional parcellation and large-scale reverse inference. *Cereb. Cortex* **23,** 739–749 (2013);

SUPPLEMENTARY TABLES S1-S9

Supplementary Table S1. Total variance explained.

Component	A. Initial eigenvalues			B. Rotation sums of squared loadings		
	Total	% of variance	Cumulative %	Total	% of variance	Cumulative %
1	4.042	25.264	25.264	2.419	15.120	15.120
2	2.305	14.404	39.667	2.299	14.371	29.492
3	1.967	12.293	51.961	2.065	12.909	42.400
4	1.480	9.248	61.209	1.922	12.010	54.410
5	1.166	7.285	68.494	1.839	11.494	65.904
6	1.025	6.403	74.897	1.439	8.993	74.897
7	.927	5.794	80.691			
8	.686	4.287	84.978			
9	.530	3.310	88.288			
10	.465	2.906	91.193			
11	.350	2.191	93.384			
12	.311	1.946	95.329			
13	.283	1.767	97.097			
14	.202	1.262	98.359			
15	.142	.886	99.244			
16	.121	.756	100.000			

The table shows the distribution of the variance (eigenvalues, percentage of total variance accounted by each component, and the relative cumulative percentage) before (A) and after (B) a Varimax rotation. Based on the Kaiser-Guttman criterion (eigenvalue > 1), we retained six components explaining 74.89% of the total variance.

Supplementary table S2. GM atrophy in AUD patients vs. healthy controls.

H	Brain region	Anatomy toolbox	x	y	z	T	K	TFCE
	Superior medial gyrus		2	24	40	**6.53**	**155**	**7210.86**
	Posterior-medial frontal		-3	4	45	**6.25**	**47**	**6851.36**
	Anterior cingulate cortex		-2	45	15	**7.37**	**310**	**7538.58**
	Rectus gyrus	Fp2	0	50	-16	**6.62**	**67**	**6835.83**
	vmPFC	s24	0	16	-9	**6.25**	**24**	**7197.77**
L	Rolandic operculum		-46	-2	3	**6.02**	**9**	**5624.14**
L	Rolandic operculum	OP2	-36	-24	15	**6.16**	**48**	**6214.18**
R	Rolandic operculum	OP1	50	-27	20	**7.15**	**321**	**6858.31**
R	Posterior insula	Ig2	38	-18	14	**6.09**		**6454.81**
R	Rolandic operculum	OP4	54	-3	6	**6.71**	**130**	**6369.91**
R	Rolandic operculum	44	62	9	12	**7.38**	**48**	**5884.92**
R	Posterior insula	Ig2	38	-16	4	**6.21**	**27**	**6408.57**
R	Anterior insula		36	24	-3	**6.11**	**48**	**5823.82**
L	Superior temporal gyrus	OP4	-52	-15	10	**6.64**	**261**	**6431.13**
L	Supramarginal gyrus	OP1	-57	-22	18	**6.09**		**6308.75**
R	Supramarginal gyrus	PFcm	60	-32	24	**6.03**	**9**	**6210.25**
L	Middle temporal gyrus		-63	-40	-14	**6.22**	**6**	**2673.78**
R	Middle temporal gyrus		54	-18	-9	**7.70**	**148**	**5758.89**
R	Inferior temporal gyrus	FG4	46	-48	-20	**6.13**	**8**	**4451.95**
L	Postcentral gyrus	2	-36	-36	51	**6.51**	**19**	**4500.84**
	Posterior cingulate cortex		0	-50	33	**7.59**	**180**	**6980.89**
R	Precuneus		3	-68	24	**6.22**	**26**	**6683.07**
R	Lingual gyrus		12	-45	-2	**6.42**	**14**	**6704.80**
R	Hippocampus (CA1)		36	-38	-6	**6.73**	**37**	**5589.93**
L	Amygdala		-14	-2	-15	**5.83**	**3**	**6316.35**
	Ventral striatum		2	2	4	**6.17**	**31**	**7197.22**
	Ventral striatum		2	9	-2	**5.87**	**15**	**7159.09**
L	Thalamus		-10	-32	8	**6.34**	**111**	**7209.67**
R	Thalamus		10	-34	6	**7.34**	**255**	**7771.35**

H: hemisphere; L: left; R: right; Fp2: medial frontopolar area 2; vmPFC: ventromedial prefrontal cortex; OP: parietal operculum; FG: fusiform gyrus; K: cluster extent in number of voxels (1x1x1 mm^3); TFCE: Threshold-Free-Cluster-Enhancement. Bold font denotes a statistically significant effect at $p<0.025$ corrected for multiple comparisons, either at voxel (T), cluster (K) or TFCE levels.

Supplementary table S3. Correlation between GM density and executive performance.

H	Brain region	Anatomy toolbox	x	y	z	T	K	TFCE
L	IFG (pars orbitalis)		-42	20	-6	**5.9**	**3755**	**2166.27**
L	IFG (pars triangularis)	45	-46	28	15	4.16		**1537.70**
L	IFG (pars opercularis)	44	-54	14	12	3.94		**1667.29**
L	Temporal pole		-50	12	-6	4.65		**1940.72**
L	Medial temporal pole		-18	8	-39	4.02		**1530.29**
L	ParaHippocampal gyrus	Amygdala (LB)	-21	2	-27	4.87		**1639.36**
L	Middle orbital gyrus	Fo3	-21	34	-18	3.97	**3495**	**1427.00**
R	IFG (pars opercularis)		51	12	-2	4.11		**1547.75**
R	Anterior insula		33	12	-18	4.16		**1564.87**
R	Hippocampus		20	-8	-14	4.29		**1676.42**
R	Caudate nucleus		8	9	-4	4.08		**1610.74**
R	Medial temporal cortex		12	-10	-15	4.95		**1719.55**
R	Ventral striatum		15	4	-16	4.89		**1756.95**
R	Rolandic operculum		52	-27	22	4.01	**872**	**1386.47**
R	Superior temporal gyrus	PFcm (IPL)	54	-33	20	3.72		**1370.38**
R	Middle temporal gyrus		46	-38	-2	3.57		**1289.01**
R	Supramarginal gyrus	PF (IPL)	63	-30	28	3.52		**1348.63**
L	Cerebellum (VIII)	Lobule VIIIa	-24	-57	-58	**6.44**	**1567**	**2231.67**

H: hemisphere; L: left; R: right; IFG: inferior frontal gyrus; LB: latero-basal amygdala nuclei; Fo3: medial orbital sulcus; IPL: inferior parietal lobule; K: cluster extent in number of voxels (1x1x1 mm^3); TFCE: Threshold-Free-Cluster-Enhancement. Bold font denotes a statistically significant effect at $p<0.025$ corrected for multiple comparisons, either at voxel (T), cluster (K) or TFCE levels.

Supplementary table S4. Correlation between GM density and TMT-A response time.

H	Brain region	Anatomy toolbox	x	y	z	T	K	TFCE
	dACC		0	38	26	4.94	**279**	**1759.94**
R	Amygdala		21	5	-17	**5.80**	**509**	**1764.37**
R	IFG (pars orbitalis)		29	12	-23	3.51		**1705.77**
L	Insula lobe		-42	9	-5	4.37	**735**	**1763.49**
L	IFG (pars orbitalis)		-48	20	-3	4.28		**1612.84**
L	Temporal pole		-42	18	-15	4.15		**1739.57**
L	Rolandic operculum		-45	-2	2	3.89		**1592.78**
L	Superior temporal gyrus	OP4	-56	-5	5	3.47		**1626.59**
R	Anterior insula		47	12	-6	4.57	**356**	**1742.75**
R	Rolandic operculum	OP4	54	-3	6	4.8	**1173**	**1805.70**
R	Rolandic operculum	OP1	50	-26	18	4.59		**1886.79**
R	Posterior insula	Ig2	39	-15	0	4.27		**1751.76**
R	Middle insula	OP3	36	-6	15	4.09		**1726.07**
R	Superior temporal gyrus	PFcm (IPL)	57	-33	23	3.92		**1790.14**
R	Posterior insula	OP2	36	-18	15	3.7		**1715.18**
L	Superior temporal gyrus		-56	-17	9	4.98	**769**	**1853.45**
L	Rolandic operculum	OP3	-38	-17	18	4.33		**1749.35**
L	Posterior insula		-33	-21	14	4.32		**1748.74**
L	Supramarginal gyrus	OP1	-59	-21	18	4.22		**1775.27**
R	Fusiform gyrus	FG4	29	-32	-26	4.57	**407**	**1864.69**
R	Lingual gyrus		12	-44	0	5.18	**917**	**1927.88**
L	Calcarine gyrus		-5	-56	3	4.28		**1828.74**
R	Precuneus		15	-53	15	4.23		**1726.30**
L	Lingual gyrus		-9	-44	0	3.52		**1706.56**
R	Calcarine gyrus		5	-59	14	3.45		**1708.25**

H: hemisphere; L: left; R: right; dACC: dorsal sector of anterior cingulate cortex; IFG: inferior frontal gyrus; OP: parietal operculum; IPL: inferior parietal lobule; FG: fusiform gyrus; K: cluster extent in number of voxels (1x1x1 mm^3); TFCE: Threshold-Free-Cluster-Enhancement. Bold font denotes a statistically significant effect at $p<0.025$ corrected for multiple comparisons, either at voxel (T), cluster (K) or TFCE levels.

Supplementary table S5. Common effects of AUDs and correlation with executive performance.

H	Brain region	Anatomy toolbox	x	y	z	T	K
L	IFG (pars orbitalis)		-42	20	-3	5.18	**2652**
L	IFG (pars triangularis)		-50	20	10	4.36	
L	IFG (pars opercularis)	44	-54	14	12	3.94	
L	Anterior insula		-46	9	-6	4.42	
L	Temporal pole		-32	8	-22	3.34	
L	Amygdala	LB	-20	3	-27	4.28	
R	Anterior insula		34	18	-18	3.61	**2355**
R	Middle insula		50	12	-2	4.09	
R	Medial temporal cortex		12	-10	-15	4.95	
R	Amygdala		16	-9	-9	4.29	
L	Ventral striatum		-6	9	-6	3.77	
R	Ventral striatum		16	3	-16	4.5	
R	Ventral striatum		8	9	-4	4.08	
R	Rolandic operculum	PFop (IPL)	52	-27	22	4.01	**648**
R	Superior temporal gyrus	PFcm (IPL)	54	-33	20	3.72	
R	Supramarginal gyrus	PF (IPL)	63	-30	28	3.52	

H: hemisphere; L: left; R: IFG: inferior frontal gyrus; LB: latero-basal amygdala nuclei; IPL: inferior parietal lobule; K: cluster extent in number of voxels (1x1x1 mm^3). Bold font denotes a statistically significant effect at $p<0.025$ corrected for multiple comparisons (note that TFCE statistics are not available for conjunction analyses).

Supplementary table S6. Common effects of AUDs and correlation with TMT-A response time.

H	Brain region	Anatomy toolbox	x	y	z	T	K
R	dACC		0	38	26	4.94	**1073**
R	Pregenual cortex		2	46	9	3.73	
R	vmPFC/subgenual cortex	s24	2	30	-8	3.02	
R	Medial temporal cortex		10	-9	-16	5.5	**3172**
R	Amygdala		21	4	-16	4.01	
R	Ventral striatum		12	3	-16	3.91	
R	vmPFC/subgenual cortex	s24	0	16	-10	3.64	
L	Superior temporal gyrus		-56	-16	9	4.98	**4594**
L	Rolandic operculum	OP3	-38	-16	18	4.33	
L	Posterior insula		-33	-21	14	4.32	
L	Middle insula		-42	9	-4	4.3	
L	IFG (pars orbitalis)		-48	20	-3	4.28	
L	Supramarginal gyrus	OP1	-58	-21	18	4.22	
L	IFG (pars opercularis)	44	-54	10	12	4.08	
R	Middle insula		46	12	-6	4.57	**1045**
R	Middle insula		40	2	-10	3.76	
R	Anterior insula		34	24	3	3.24	
R	Rolandic operculum	OP4	54	-3	6	4.8	**3208**
R	Rolandic operculum	OP1	50	-26	18	4.59	
R	Posterior insula	Ig2	39	-15	0	4.27	
R	Frontal operculum	44	62	9	12	4.11	
R	Posterior insula	OP3	36	-6	15	4.09	
R	Superior temporal gyrus	PFcm (IPL)	57	-33	22	3.92	
R	Inferior temporal gyrus		58	-24	-21	4.35	**609**
R	Middle temporal gyrus		56	-20	-10	3.84	
R	Superior temporal gyrus		56	-32	3	3.3	
R	Lingual gyrus		12	-44	0	5.18	**3173**
R	Posterior hippocampus		38	-36	-6	4.83	
L	Lingual Gyrus		-6	-54	3	4.27	
R	Cerebellar Vermis (4/5)		4	-56	4	3.99	
L	Cerebellum (IV-V)		-8	-39	-3	3.65	

H: hemisphere; L: left; R: right; dACC: dorsal sector of anterior cingulate cortex; vmPFC: ventromedial prefrontal cortex; IFG: inferior frontal gyrus; OP: parietal operculum; IPL: inferior parietal lobule; K: cluster extent in number of voxels (1x1x1 mm^3). Bold font denotes a statistically significant effect at $p<0.025$ corrected for multiple comparisons (note that TFCE statistics are not available for conjunction analyses).

Supplementary Table s7. Correlation Matrix.

	DS	IR	DR	IM-10	IM-30	TMT-A	TMT-B	TT	PF	AVR	CE	OF	CD	SD	CLD	PA
DS	1.000	.211	.100	.303	.011	-.171	-.188	-.300	.180	.152	.394	.157	.140	-.136	-.140	-.033
IR	.211	1.000	.787	.344	.171	-.369	-.448	.139	.088	.245	.118	.448	.151	.447	.365	.173
DR	.100	.787	1.000	.301	-.005	-.224	-.450	.344	.083	.151	.219	.333	.161	.363	.301	.047
IM-10	.303	.344	.301	1.000	.396	-.390	-.250	-.165	.089	.038	-.028	.252	-.002	.121	.089	.076
IM-30	.011	.171	-.005	.396	1.000	-.343	.100	-.084	.133	.008	-.170	.274	-.146	.299	.176	.279
TMT-A	-.171	-.369	-.224	-.390	-.343	1.000	.466	-.085	.119	-.104	-.024	-.525	-.104	-.152	-.283	-.005
TMT-B	-.188	-.448	-.450	-.250	.100	.466	1.000	-.164	.107	-.184	-.353	-.391	-.397	-.072	-.178	.092
TT	-.300	.139	.344	-.165	-.084	-.085	-.164	1.000	-.512	-.117	.226	.231	.037	.292	.087	-.044
PF	.180	.088	.083	.089	.133	.119	.107	-.512	1.000	.013	-.087	-.046	.106	-.080	.146	.107
AVR	.152	.245	.151	.038	.008	-.104	-.184	-.117	.013	1.000	.055	.207	.280	.127	.066	.295
CE	.394	.118	.219	-.028	-.170	-.024	-.353	.226	-.087	.055	1.000	.159	.403	.093	-.078	-.086
OF	.157	.448	.333	.252	.274	-.525	-.391	.231	-.046	.207	.159	1.000	.359	.479	.512	.353
CD	.140	.151	.161	-.002	-.146	-.104	-.397	.037	.106	.280	.403	.359	1.000	-.124	.096	-.086
SD	-.136	.447	.363	.121	.299	-.152	-.072	.292	-.080	.127	.093	.479	-.124	1.000	.469	.629
CLD	-.140	.365	.301	.089	.176	-.283	-.178	.087	.146	.066	-.078	.512	.096	.469	1.000	.576
PA	-.033	.173	.047	.076	.279	-.005	.092	-.044	.107	.295	-.086	.353	-.086	.629	.576	1.000

The table reports the cross-correlation coefficients among the 15 tests of the Brief Neuropsychological Examination (ENB2). DS = Digit span, IR = Immediate recall, DR= Delayed recall, IM-10 = Interference memory 10”, IM-30 = Interference memory 30”, TMT-A = Trial Making test A, TMT-B = Trial Making test B, TT = Token test, PF = Phonemic fluency, AVR = Abstract verbal reasoning, CE = cognitive estimation, OF = Overlapping figures, CD = copy drawing, SD = Spontaneous drawing, CLD = Clock drawing, PA = Praxis abilities.

Supplementary Table S8. Communalities.

	Communalities
Digit span	.795
Immediate recall	.823
Delayed recall	.906
Interference memory 10’’	.682
Interference memory 30’’	.656
Trial Making test A	.792
Trial Making test B	.702
Token test	.809
Phonemic fluency	.754
Abstract verbal reasoning	.411
Cognitive estimation	.755
Overlapping figures	.752
Copy drawing	.743
Spontaneous drawing	.835
Clock drawing	.712
Praxis abilities	.856

For each variable of the Brief Neuropsychological Examination (ENB2), the table shows the proportion of variance explained by the retained components after the Varimax rotation.

Supplementary Table S9. Rotated component matrix.

	Component					
	1	2	3	4	5	6
Digit span	**.916**					
Immediate recall	**.791**	.334				
Delayed recall	**.619**					-.387
Interference memory 10’’		**.936**				
Interference Memory 30’’		**.823**				
Trial Making test A			**-.779**	-.345		
Trial Making test B			**.711**			
Token test	.338		**.686**			
Phonemic fluency				**.839**		
Abstract verbal reasoning		-.466		**-.592**		
Cognitive estimation	.472		.439	**.496**		
Overlapping figures	.352			**.458**		
Copy drawing					**.844**	
Spontaneous drawing					**-.830**	
Clock drawing						**.791**
Praxis abilities				.315		**.716**

The table shows the correlations between the ENB variables and the estimated components after the Varimax rotation. Correlation coefficients < 0.3 are not reported.

Supplementary Figure 1.

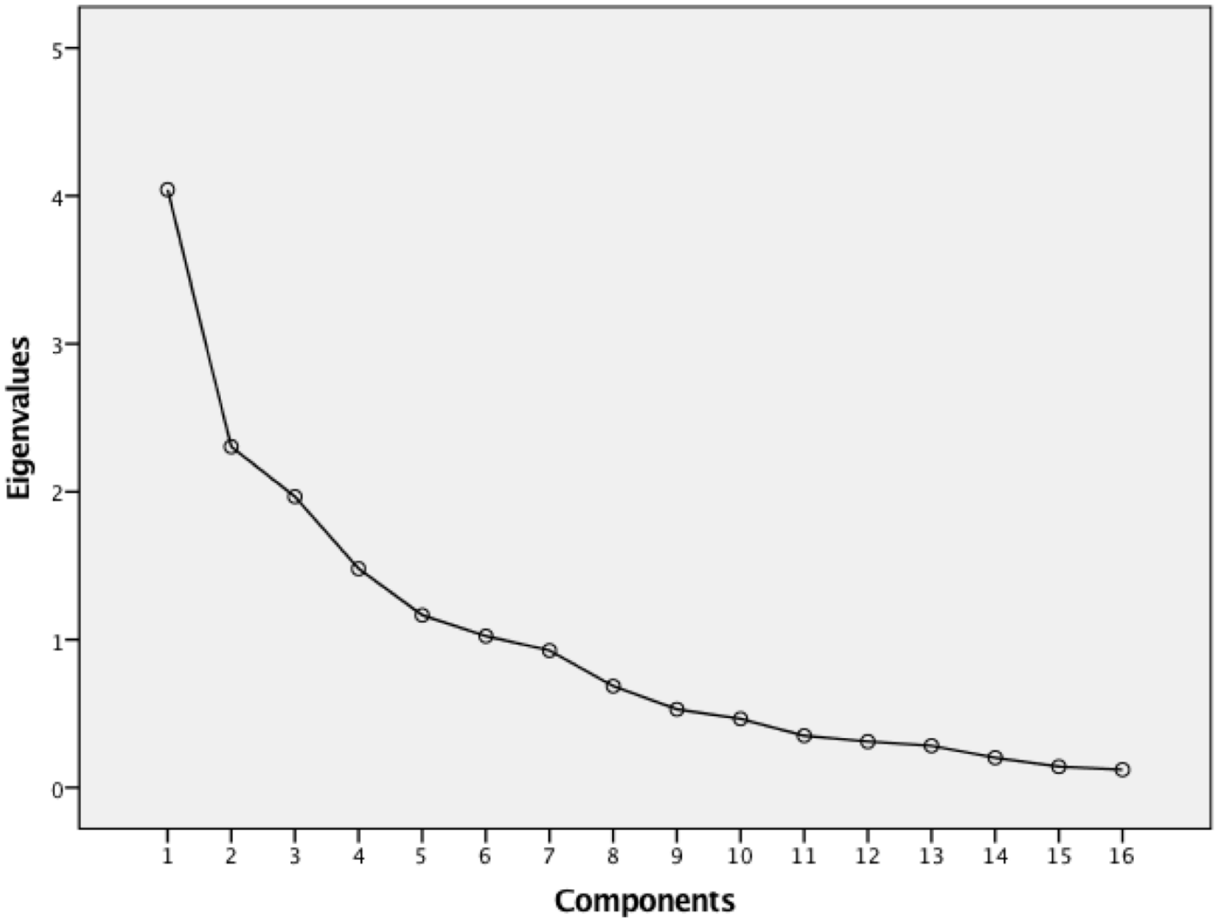

Supplementary Figure 1. The figure shows the scree plot of the principal component analysis performed on the 15 scores of the Brief Neuropsychological Examination (ENB2).
